# Assessment of Vaginal Lactobacillary Flora in Wet Mount and Fresh or Delayed Gram's Stain

**DOI:** 10.1155/S1064744996000026

**Published:** 1996

**Authors:** Gilbert G. G. Donders, Annie Vereecken, Geert Salembier, Ben Van Bulck, Bernard Spitz

**Affiliations:** 1 Department of Obstetrics and Gynecology University Hospital Gasthuisberg Katholieke Universiteit Leuven Herestraat 49 Leuven 3300 Belgium; 2 Laboratory of Clinical Pathology Division of Microbiology Tolstraat Antwerp Belgium; 3 Department of Gynecology Erasmus Hospital Antwerp Belgium

## Abstract

*Objective:* The assessment of the vaginal lactobacillary flora helps to direct further diagnostic microbiologic investigations in genital infectious disease and seems to represent a powerful tool in predicting infectious morbidity and preterm labor during pregnancy. In the absence of a “gold standard,” we studied the variations in assessing lactobacillary morphotypes according to the method used.

*Methods:* The lactobacillary flora from 183 pregnant women was classified according to 3 groups: normal, intermediate, and abnormal. This grading of lactobacilli was appled to vaginal and cervical specimens by means of 1) immediate wet-smear microscopy, 2) Gram's stain on a fresh, air-dried specimen, and 3)delayed Gram's stain after specimen transportation in Stuart's growth medium for 3–6 h.

*Results:* The assignment of intermediate or abnormal flora (grade II or grade III) showed high concordance rates among the different preparatory techniques, but the assignment of grade I (normal flora) did not. Fewer lactobacilli were found 2.6 times more often after Gram's stains of fresh specimens [Relative Risk (RR) 2.6, 95% confidence interval (CI) 1.7–4.1] and 6 times more often when the Gram%s stain was performed in a delayed examination after transport than in a fresh wet-mount specimen (RR 6.2, 95% CI 2.5–15.6). Disturbed lactobacillary grades were also found more frequently in specimens from the cervix than those from the vagina (RR 4.0, 95% CI, 1.5–10.4).

*Conclusions:* There are discrepancies in the diagnosis of lactobacillary grades between gram-stained and fresh vaginal specimens. The evidence is ambiguous as to which of the 2 methods is responsible. If an evaluation is to be done on a gram-stained specimen, then the storage of the sample in Stuart transport medium before staining should be avoided.


Infectious Diseases in Obstetrics and Gynecology 4:2–6 (1996)
© 1996 Wiley-Liss, Inc.

### Assessment of Vaginal Lactobacillary Flora in Wet Mount and Fresh or Delayed Gram's Stain

Gilbert G.G. Donders, Annie Vereecken, Geert Salembier, Ben Van Bulck, and Bernard Spitz

*Department of Obstetrics and Gynecology, University Hospital Gasthuisberg, Katholieke Universiteit Leuven, Leuven (G.G.G.D., B.S.); Laboratory of Clinical Pathology, Division of Microbiology, Tolstraat, Antwerp (A.V., G.S.); and Department of Gynecology, Erasmus Hospital, Antwerp (B.V.B.), Belgium*

ABSTRACT

***Objective:*** **The assessment of the vaginal lactobacillary flora helps to direct further diagnostic microbiologic investigations in genital infectious disease and seems to represent a powerful tool in predicting infectious morbidity and preterm labor during pregnancy. In the absence of a "gold standard," we studied the variations in assessing lactobacillary morphotypes according to the method used.**

***Methods:*** **The lactobacillary flora from 183 pregnant women was classified according to 3 groups: normal, intermediate, and abnormal. This grading of lactobacilli was appled to vaginal and cervical specimens by means of 1) immediate wet-smear microscopy, 2) Gram's stain on a fresh, air-dried specimen, and 3) delayed Gram's stain after specimen transportation in Stuart's growth medium for 3–6 h.**

***Results:*** **The assignment of intermediate or abnormal flora (grade II or grade III) showed high concordance rates among the different preparatory techniques, but the assignment of grade I (normal flora) did not. Fewer lactobacilli were found 2.6 times more often after Gram's stains of fresh specimens [Relative Risk (RR) 2.6, 95% confidence interval (CI) 1.7–4.1] and 6 times more often when the Gram's stain was performed in a delayed examination after transport than in a fresh wet-mount specimen (RR 6.2, 95% CI 2.5–15.6). Disturbed lactobacillary grades were also found more frequently in specimens from the cervix than those from the vagina (RR 4.0, 95% CI, 1.5–10.4).**

***Conclusions:*** **There are discrepancies in the diagnosis of lactobacillary grades between gram-stained and fresh vaginal specimens. The evidence is ambiguous as to which of the 2 methods is responsible. If an evaluation is to be done on a gram-stained specimen, then the storage of the sample in Stuart transport medium before staining should be avoided.** © 1996 Wiley-Liss, Inc.

KEY WORDS

Pap smear, lactobacillary grades, bacterial vaginosis

Since its discovery by Döderlein[1] in 1892, the vaginal *Lactobacillus* has fascinated researchers and clinicians with its intriguing ability to protect women from a large number of pathogens entering the upper genital tract. Schröder[2] (1913) and Hunter[3] (1945) divided the vaginal flora into 3 subtypes in order to link them to clinical vaginitis and vaginal pathogens. However, they were limited by the diagnostic facilities available at that time and by unknown microorganisms yet to be discovered.

Only after the discovery of bacterial vaginosis ("non-specific vaginitis")[4] and its etiopathogenic relationship with the vaginal lactobacilli[5] did the lactobacillary flora regain attention as an antimicrobial defense mechanism of the vagina. The peroxidase-producing abilities of lactobacilli, being decreased in bacterial vaginosis,[6] may play a key role in the vaginal defense system.

In addition to bacterial vaginosis, usually caused by *Gardnerella vaginalis*, *Bacteroides* sp., and *Mobilun-*

Address reprint requests to Dr. Gilbert G.G. Donders, Department of Obstetrics and Gynecology, University Hospital Gasthuisberg, Katholieke Universiteit Leuven, Herestraat 49, 3300 Leuven, Belgium.

Received January 15, 1996
Accepted May 6, 1996

Clinical Study

*cus* sp., disturbed lactobacillary morphotypes have been linked to genital *Chlamydia* infection,[7] gonorrhea,[8] *Trichomonas vaginalis* infection,[8] and vaginitis caused by aerobes.[9] Decreased lactobacillus numbers are associated with increased risks of preterm delivery, low birth weight,[8,10,11] and midtrimester pregnancy loss.[10] It should be noted that a disturbed lactobacillary flora (grade III), although frequently associated, is not diagnostic of bacterial (anaerobic) vaginosis.

The evaluation of a genital infection and routine screening during pregnancy should always include a careful assessment of the vaginal lactobacillary flora. Studies have shown a relation between the absence of lactobacillary morphotypes and low birth weight and preterm birth.[8,10] Further investigation may lead to the diagnosis of bacterial vaginosis, *C. trachomatis, Neisseria gonorrhoeae, T. vaginalis*, or aerobic vaginitis,[7,8,11] enabling efficient treatment during pregnancy. However, not only may the assessment of lactobacillary morphotypes vary according to the method used, a "gold standard" is lacking. Lactobacillary grades[2,12] and the diagnosis of bacterial vaginosis[5] are usually based on gram-stained specimens or Pap smears,[8] the reasons for which lie more in the conditioned reflexes of many clinicians to order laboratory tests and their lack of sufficient experience in performing microscopy than in the accuracy of the fresh vaginal smear (wet mount) itself. Still, in clinical practice, the microscopic examination of a wet mount is the most practical tool because it directs further diagnostic investigations without delay and enables immediate results and prompt adequate treatment.

In our study, we semiquantitatively compared the lactobacillary morphotypes in fresh vaginal wet mounts with those in gram-stained specimens. In addition, we studied the influnces of swab transport in Stuart medium and the site of sampling (cervix or vaginal vault) on the vaginal lactobacillary flora.

The results of our study should raise some questions about the validity of the Gram's stain as a method of choice in evaluating a genital infection and help in improving the standardization of the procedures used.

### MATERIALS AND METHODS

### Subjects and Specimen Sampling

From January 1992 to December 1993, 183 consecutive patients presenting for routine pregnancy checkup or for presumed genital infection underwent standardized vaginal speculum examinations. An unmoistened speculum was inserted before any other vaginal examination was performed. Vaginal fluid was taken from the posterior vaginal vault with a wooden Ayre's spatula and spread on 2 separate glass slides. On the first glass slide, a drop of 0.9% NaCl in water was applied and covered with a glass slip for immediate microscopic evaluation. The other glass slide was air-dried for a Gram's stain to be performed later.

Subsequently, a sample was taken from the posterior vaginal vault with a cotton-tipped swab and immediately placed in Amies' modified Stuart medium. After the ectocervix was cleaned, an endocervical swab was allowed to soak for 20 seconds in the endocervical canal, rotated 3 times, and placed in a second Amies' modified Stuart medium. Both swabs and the air-dried glass slide were transported to the laboratory for Gram's stain within 6 h.

### Grading of the Lactobacillary Morphotypes

The wet mount was classified according to Schröder's original classification.[2] This grading system was chosen because of its value in clinical practice during pregnancy.[8,10] Normal, grade-I flora corresponds to predominantly lactobacillary morphotypes, with very few coccoid bacteria present. Care must be taken not to identify the cellular debris from lysed epithelial cells as coccoid bacteria. The intermediate, grade-II flora corresponds to a diminished lactobacillary flora, which is mixed with other bacteria. Finally, the abnormal, grade-III flora consists of numerous other bacteria, with no lactobacilli present.

The two swabs (vaginal and endocervical) that had been placed in Amies' modified Stuart medium at room temperature for 3–6 h were each rolled over a glass slide, fixed in pure alcohol, and stained according to the Gram's method.[5] A third glass slide that had been prepared immediately after sampling was allowed to dry in air for the same 3–6 h before being gram stained in the same manner. The slides were evaluated for lactobacillary grade by one trained technician who was blind to the origin of the slides and to the culture results.

### Statistical Analysis

The grades assigned to 2 slides that have been treated differently could be concordant or discordant.

**TABLE 1. Comparison of lactobacillary (LB) grades according to fixation method, transport medium, and site of sampling**

| | Fixation method (vaginal wet mount vs. Gram's stain) | | | | Stuart transport medium (vaginal Gram's stain fresh vs. after transport) | | | | Site of sampling (Gram's stain from vagina vs. cervix) | | | |
|---|---|---|---|---|---|---|---|---|---|---|---|---|
| | N | Concordant LB grade | Lower LB grade | Higher LB grade | N | Concordant LB grade | Lower LB grade | Higher LB grade | N | Concordant LB grade | Lower LB grade | Higher LB grade |
| Grade I | 73 | 25 (35%) | — | 48 | 38 | 11 (29%) | — | 27 | 20 | 7 (35%) | — | 13 |
| Grade II | 66 | 47 (71%) | 12 | 7 | 106 | 99 (93%) | 3 | 4 | 131 | 123 (94%) | 1 | 7 |
| Grade III | 29 | 20 (69%) | 9 | — | 26 | 24 (92%) | 2 | — | 32 | 28 (88%) | 4 | — |
| Total | 168 | 92 (55%) | 21 (12%) | 55 (33%) | 170 | 134 (79%) | 5 (2.9%) | 31 (18%) | 183 | 158 (86%) | 5 (3%) | 20 (11%) |

We first calculated whether the grades were equally distributed in fresh and in gram-stained specimens and whether the lactobacillary expression was systematically either overestimated or underestimated after gram staining. To this end, the lactobacillary gradings for fresh vaginal smears were compared with the gradings for gram-stained, air-dried slides (comparison group A). The hypothesis to be tested was that lactobacilli are equally well detected in fresh as in gram-stained specimens.

Second, we studied whether keeping the swab in Stuart modified medium for up to 6 h influenced the lactobacillary flora. The grades after gram staining of fresh vaginal smears were compared with vaginal swabs from the transport medium (comparison group B).

Finally, we tested the hypothesis that the gram-stained slide from a cervical swab is more likely to reveal a higher lactobacillary grade (reflecting the presence of fewer lactobacilli) than one from a vaginal swab (comparison group C), both having been transported in Stuart medium. As vaginal glycogen is of importance for their growth, more lactobacilli were expected to be found in the vagina than in the cervix.[13,14]

The Fisher's test was used for analysis of 2 × 2 tables, and the relative risk (RR) with 95% confidence interval (CI) was calculated to express any significant decrease in lactobacilli in the various groups.

### RESULTS

Overall, the concordance rates for the different comparisons varied from 55% for comparison group A (wet mount vs. Gram's stain of fresh specimen) to 79% for comparison group B (Gram's stain of fresh vs. delayed) to 86% for comparison group C (Gram's stain of vagina vs. cervix) (Table 1). However, the distribution of the grades within the groups varied. When the figures were standardized for the contribution of the lactobacillary grades, the overall concordance rates were similar in comparison group A (55%), comparison group B (62%), and comparison group C (67%, $P > 0.05$).

The influence of the preparation technique was more striking for the assignment of grade-I lactobacillary flora than for the observation of disturbed flora. For the assignment of grade I, there was only 29% concordance between fresh and delayed Gram's stain (comparison group B) and 35% concordance when wet mount was compared with Gram's stain (comparison group A) and when the vaginal swab was compared with the cervical swab (comparison group C). The assignment of grade II or III showed high concordance rates in both comparison group B and comparison group C (88–94%), but lower concordance in comparison group A (69–71%).

When the assignment of grades within each group was analyzed, higher lactobacillary grades (fewer lactobacilli) were found 2.6 times more frequently after Gram's stain than in fresh specimens (RR 2.6, 95% CI 1.7–4.1, $P < 0.001$). Similar differences were found when the lactobacillary flora by gram-stained smears after transport in Stuart medium compared with the assessment of fresh gram-stained specimens (RR 6.2, 95% CI 2.5–15.6, $P < 0.0001$). Higher lactobacillary grades were also more frequently found in specimens from the cervix compared with those from the vagina (RR 4.0, 95% CI 1.5–10.4, $P < 0.005$).

### DISCUSSION

The microscopic appearance of the vaginal lactobacillary flora, an indirect measure of the presence of pathogenic microorganisms in the vagina or cervix, may be linked to an increased risk of premature delivery in pregnant women.[8,10] There is, however, the question of whether the mode of assessment (different clinical settings, different sampling techniques, or different methods of preparation for microscopy) has an influence on the grade assigned to the vaginal lactobacillary flora in a specimen.

In the comparison of wet mount and gram staining, the concordance rates before and after gram staining were high when the vaginal lactobacillary flora was moderately or severely disturbed (grades II and III), but low when the flora was normal (grade I). The specimens assessed as grade I by wet mount were frequently assigned to a higher grade after gram staining. The reason for such discordance could be either a false overestimation of the proportion of lactobacilli in the wet mount of a false underestimation in the gram-stained specimen. In support of the first premise, certain bacteria may have been erroneously classified as lactobacillary morphotypes on wet mount and correctly identified as nonlactobacillary, e.g., diphtheroids, after gram staining. This error in the wet-mount method would be particularly pronounced in grade-I specimens in which the proportion of lactobacillary morphotypes is high.

The discordance could also be an artifact of the staining technique: lactobacilli may adhere less strongly than nonlactobacilli to the glass plate and the vaginal epithelial cells during the staining procedure.[15,16] *Escherichia coli* and streptococci are more adhesive to foreign material than are lactobacilli[17] and *G. vaginalis* is known to stick to epithelial cells more persistently than do lactobacilli.[18] The staining artifact, based on selective wash-out of lactobacilli, would have its most pronounced effect in grade-I specimens, in which the proportions of lactobacilli are highest.

The premise that the Gram's stain can lead to an improved diagnosis of an abnormal lactobacillary grade is supported by the fact that clue cells, normally associated with grade-III flora in bacterial vaginosis, were seen in 12% of the specimens from asymptomatic women after gram staining, whereas, on wet mounts, they were seen in only 4.5% of such specimens.[19]

The phenomenon of selective wash-out of lactobacilli suggested for gram staining may also be a problem with Pap smears. In one of our previous studies, we used lactobacillary grading on Pap smears as a prescreening tool for detecting women at high risk of genital infection.[8] In an unselected group of women presenting for routine prenatal care, the prevalence of grade III was unexpectedly high (48%). Although we realized that only a selected number of microorganisms was evaluated in our study, 46% of these grade-III scores remained unexplained, thereby greatly reducing the specificity of this method of screening. Perhaps also during the preparation of a Pap smear, the lactobacilli are selectively washed out, producing false-positive results for the lactobacillary grade.

The results from the comparison of gram staining with wet mounts showed a further discrepancy in that, of those specimens assessed as grade III (no lactobacilli) on wet mount, 30% revealed lactobacilli on gram staining. Bacterial adherence is known to be pH-dependent. A grade-III flora is usually associated with an increased pH. It is known that *Gardnerella* adheres less strongly at a high than at a low pH.[15,18] Thus, the picture of variable adherence characteristics described above for grade-I specimens may be reversed to some extent in grade-III samples. During the staining procedure, nonlactobacilli may be more easily washed away from grade-III specimens (high pH) than they were from grade-I specimens (low pH), thus revealing the very few lactobacilli present but masked in the wet mount by the high numbers of nonlactobacilli.

The discordances could also be partially due to misidentification. In fresh specimens, the small, coarse gram-positive lactobacilli may have been mistaken for pathogenic gram-negative bacteria such as *E. coli*, *Klebsiella* sp., *Mobiluncus*, or *G. vaginalis*. The motility of *Mobiluncus*, being lost on staining, may result in the misidentification of this bacterium as a coarse type of lactobacillus.[20]

When the results of the freshly gram-stained vaginal smears were compared with those after remaining in transport medium for 3–6 h, the concordance rates were very good for specimens assessed as grade II (93%) or III (92%), but were poor for those assessed as grade I (29%). We conclude that keeping the swab in the Stuart transport medium caused a selective decrease in the number of lactobacilli. Therefore, in the assessment of lactobacillary grades, specimens to be gram stained should be handled immediately and the use of transport medium should be avoided.

The actual periods of residence in the transport medium varied from 3–6 h, with the majority being processed 5 h after sampling. The time differences were thus insufficient for a time-lag analysis. However, a study of the progressive influence of Stuart medium on the growth of lactobacilli vs. nonlactobacilli over time might be of considerable interest.

Finally, the sampling site also appeared to influence the lactobacillary grade assigned to the bacterial flora. Endocervical specimens revealed lower numbers of lactobacilli than did vaginal smears, which might be explained by the physiologic bacteriostatic properties of the endocervical mucus and its higher pH. For the evaluation of lactobacillary grades, the endocervical canal is not an adequate sampling site, as its flora does not reflect that present in the vagina.

We conclude that discrepancies in the diagnosis of lactobacillary grades occur between gram-stained and fresh specimens. The evidence is ambiguous as to which of the 2 methods is responsible. If an evaluation is to be carried out on gram-stained specimens, the samples should not be stored in Stuart transport medium before staining.

### ACKNOWLEDGMENTS

We thank Mrs. Alison Odds and Prof. M Hanssens for the critical review of the manuscript.

### REFERENCES

1. Döderlein A: Das Sdheidensekret und seine Bedeutung für das Puerperalfieber. Leipzig: Verlag van Eduard Besold, 1892.
2. Schröder K: Zur pathogenese und Klinik des vaginalen Vaginalbiocoenose uaf sechs grundbilder. Zentralbl Gynaekol 45:1350–1361, 1921.
3. Hunter CA, Long KR: A study of the microbiological flora of the vagina. Am J Obstet Gynecol 75:865–871, 1958.
4. Gardner HL, Dukes CD: *Haemophilus vaginalis* vaginitis. A newly defined specific infection previously classified "nonspecific" vaginitis. Am J Obstet Gynecol 69:962–976, 1955.
5. Spiegel CA, Amsel R, Holmes KK: Diagnosis of bacterial vaginosis by direct Gram stain of vaginal fluid. J Clin Microbiol 18:170–177, 1983.
6. Eschenbach DA, Davick PR, Williams BL, Klebanoff SJ, Young-Smith K, Critchlow CM, Holmes KK: Prevalence of hydrogen peroxide-producing *Lactobacillus* species in normal women with bacterial vaginosis. J Clin Microbiol 27:251–256, 1989.
7. Donders GGG, Moerman P, De Wet GH, Hooft P, Goubau P: The association between *Chlamydia* cervicitis, chorioamnionitis and neonatal complications. Arch Gynecol Obstet 249:79–85, 1991.
8. Donders GGG, De Wet GH, Hooft P, Desmyter J: Lactobacilli in Papanicolaou smears, genital infections and pregnancy. Am J Perinatol 10:358–361, 1993.
9. Gray LA, Barnes ML: Vaginitis in women, diagnosis and treatment. Am J Obstet Gynecol 92:125–136, 1965.
10. Hay PE, Lamont RF, Taylor-Robinson D, Morgan DJ, Ison C, Pearson J: Abnormal bacterial colonisation of the genital tract and subsequent preterm delivery and late miscarriage. Br Med J 308:295–298, 1994.
11. Martius J, Krohn MA, Hillier SL, et al.: Relationships of vaginal *Lactobacillus* species, cervical *Chlamydia trachomatis* and bacterial vaginosis to preterm birth. Obstet Gynecol 71:89–95, 1988.
12. Hillier SJ, Krohn MA, Nugent RP, Gibbs RS: Characteristics of three vaginal flora patterns assessed by Gram stain among pregnant women. Am J Obstet Gynecol 166:938–944, 1992.
13. Lindner JGEM, Plantema FHF, Hoogkamp-Korstanje JAA: Quanitative studies of the vaginal flora of healthy women and of obstetric and gynaecological patients. J Med Microbiol 11:233–241, 1978.
14. Cruickskank R, Sharman A: The biology of the vagina in the human subject. II. The bacterial flora and secretion of the vagina in relation to glycogen in the vaginal epithelium. J Obstet Gynaecol Br Emp 208:208–212, 1939.
15. Sobel JD, Schneider J, Kaye D, Levison ME: Adherence of bacteria to vaginal cells at various times in the menstrual cycle. Infect Immun 32:194–197, 1981.
16. Domingue PAG, Sadhu K, Costerson JW, Barlett K, Chow AW: The human vagina: Normal flora considered as an in situ tissue-associated, adherent film. Genitourin Med 67:226–231, 1991.
17. Ofek I, Beachey EH: Mannose binding and epithelial cell adherence of *Escherichia coli*. Infect Immun 22:247–251, 1978.
18. Mardh P-A, Westtom L: Adherence of bacteria to vaginal epithelial cells. Infect Immun 13:661–666, 1976.
19. Dunkelberg WE Jr: Diagnosis of *Hamophilus vaginalis* by gram-stained smears. Am J Obstet Gynecol 91:998–1000, 1965.
20. Pahlson C, Larsson PG: The ecologically wrong lactobacilli. Med Hypothesis 36:126–130, 1991.
